# A systematic review and an individual patient data meta-analysis of ivermectin use in children weighing less than fifteen kilograms: Is it time to reconsider the current contraindication?

**DOI:** 10.1371/journal.pntd.0009144

**Published:** 2021-03-17

**Authors:** Podjanee Jittamala, Wuelton Monteiro, Menno R. Smit, Belen Pedrique, Sabine Specht, Carlos J. Chaccour, Céline Dard, Pascal Del Giudice, Virak Khieu, Annabel Maruani, Virgilio E. Failoc-Rojas, Marimar Sáez-de-Ocariz, Antoni Soriano-Arandes, Jaime Piquero-Casals, Anne Faisant, Marie-Pierre Brenier-Pinchart, David Wimmersberger, Jean T. Coulibaly, Jennifer Keiser, Franck Boralevi, Oliver Sokana, Michael Marks, Daniel Engelman, Lucia Romani, Andrew C. Steer, Lorenz von Seidlein, Nicholas J. White, Eli Harriss, Kasia Stepniewska, Georgina S. Humphreys, Kalynn Kennon, Philippe J. Guerin, Kevin C. Kobylinski

**Affiliations:** 1 Department of Tropical Hygiene, Faculty of Tropical Medicine, Mahidol University, Bangkok, Thailand; 2 Mahidol-Oxford Tropical Medicine Research Unit, Faculty of Tropical Medicine, Mahidol University, Bangkok, Thailand; 3 Fundação de Medicina Tropical Dr. Heitor Vieira Dourado, Manaus, Brazil; 4 Universidade do Estado do Amazonas, Manaus, Brazil; 5 Amsterdam Centre for Global Child Health, Emma Children’s Hospital, Amsterdam, The Netherlands; 6 University Medical Centres, University of Amsterdam, Amsterdam, The Netherlands; 7 Malaria Epidemiology Unit, Department of Clinical Sciences, Liverpool School of Tropical Medicine, Liverpool, United Kingdom; 8 Drugs for Neglected Diseases *initiative* (DND*i*), Geneva, Switzerland; 9 ISGlobal, Hospital Clínic—Universitat de Barcelona, Barcelona, Spain; 10 Centro de Investigação em Saúde de Manhiça, Maputo, Mozambique; 11 Ifakara Health Institute, Ifakara, United Republic of Tanzania; 12 Instituto de Medicina Tropical Universidad de Navarra, Pamplona, Spain; 13 Laboratoire de Biologie Médicale, Centre Hospitalier de Basse-Terre, Basse-Terre, Guadeloupe, France; 14 Infectious Diseases and Dermatology Unit, Hospital of Fréjus-Saint-Raphaël, Fréjus, France; 15 National Centre for Parasitology, Entomology and Malaria Control, Ministry of Health, Phnom Penh, Cambodia; 16 Unit of Pediatric Dermatology, University Hospital Center of Tours, University of Tours, Tours, France; 17 Unidad de investigacion para la generacion y sintesis de evidencias en salud, Universidad San Ignacio de Loyola, Lima, Peru; 18 Laboratorio de Parasitologia, Metaxenicas y Zoonosis, Hospital Regional Lambayeque, Lambayeque, Peru; 19 Department of Dermatology, National Institute of Pediatrics, Mexico City, Mexico; 20 Pediatric Infectious Diseases and Immunodeficiencies Unit, Hospital Universitari Vall d’Hebron, Vall d’Hebron Research Institute, Barcelona, Spain; 21 Dermik, Multidisciplinary Dermatology Clinic, Barcelona, Spain; 22 Department of Pediatrics, University Hospital Grenoble-Alpes, Grenoble, France; 23 Departement of Parasitology-Mycology, University Hospital Grenoble-Alpes, Grenoble, France; 24 Department of Medical Parasitology and Infection Biology, Swiss Tropical and Public Health Institute, University of Basel, Basel, Switzerland; 25 Unité de Formation et de Recherche Biosciences, Université Félix Houphouët-Boigny, Abidjan, Côte d’Ivoire; 26 Centre Suisse de Recherches Scientifiques en Côte d’Ivoire, Abidjan, Côte d’Ivoire; 27 Paediatric Dermatology Unit, Children hospital, Bordeaux University Hospital, Bordeaux, France; 28 Centre d’investigation clinique pédiatrique 1401 module plurithématique, Bordeaux University, Bordeaux, France; 29 Ministry of Health and Medical Services, Honiara, Solomon Islands; 30 Clinical Research Department, Faculty of Infectious and Tropical Diseases, London School of Hygiene & Tropical Medicine, London, United Kingdom; 31 Hospital for Tropical Diseases, London, United Kingdom; 32 Tropical Diseases, Murdoch Children’s Research Institute, Melbourne, VIC, Australia; 33 Department of Paediatrics, University of Melbourne, Melbourne, VIC, Australia; 34 The Kirby Institute, UNSW, Sydney, NSW, Australia; 35 Centre for Tropical Medicine and Global Health, Nuffield Department of Clinical Medicine, University of Oxford, United Kingdom; 36 The Knowledge Centre, Bodleian Health Care Libraries, University of Oxford, Oxford, United Kingdom; 37 WorldWide Antimalarial Resistance Network (WWARN), Oxford, United Kingdom; 38 Green Templeton College, University of Oxford, United Kingdom; 39 Infectious Diseases Data Observatory (IDDO), Oxford, United Kingdom; 40 Department of Entomology, Armed Forces Research Institute of Medical Sciences, Bangkok, Thailand; Weill Cornell Medical College, UNITED STATES

## Abstract

**Background:**

Oral ivermectin is a safe broad spectrum anthelminthic used for treating several neglected tropical diseases (NTDs). Currently, ivermectin use is contraindicated in children weighing less than 15 kg, restricting access to this drug for the treatment of NTDs. Here we provide an updated systematic review of the literature and we conducted an individual-level patient data (IPD) meta-analysis describing the safety of ivermectin in children weighing less than 15 kg.

**Methodology/Principal findings:**

A systematic review was conducted using the Preferred Reporting Items for Systematic Reviews and Meta-Analyses (PRISMA) for IPD guidelines by searching MEDLINE via PubMed, Web of Science, Ovid Embase, LILACS, Cochrane Database of Systematic Reviews, TOXLINE for all clinical trials, case series, case reports, and database entries for reports on the use of ivermectin in children weighing less than 15 kg that were published between 1 January 1980 to 25 October 2019. The protocol was registered in the International Prospective Register of Systematic Reviews (PROSPERO): CRD42017056515. A total of 3,730 publications were identified, 97 were selected for potential inclusion, but only 17 sources describing 15 studies met the minimum criteria which consisted of known weights of children less than 15 kg linked to possible adverse events, and provided comprehensive IPD. A total of 1,088 children weighing less than 15 kg were administered oral ivermectin for one of the following indications: scabies, mass drug administration for scabies control, crusted scabies, cutaneous larva migrans, myiasis, pthiriasis, strongyloidiasis, trichuriasis, and parasitic disease of unknown origin. Overall a total of 1.4% (15/1,088) of children experienced 18 adverse events all of which were mild and self-limiting. No serious adverse events were reported.

**Conclusions/Significance:**

Existing limited data suggest that oral ivermectin in children weighing less than 15 kilograms is safe. Data from well-designed clinical trials are needed to provide further assurance.

## Author summary

Oral ivermectin is a safe and efficacious drug for the treatment of neglected tropical diseases. To date, ivermectin is not indicated in children weighing less than 15 kg because there have been insufficient safety data to support a change of recommendation. A PRISMA-level systematic review was conducted, and 97 potential sources were identified. All lead investigators were contacted to share individual patient data if they could provide the minimum criteria. These were the known weights of the children less than 15 kg in whom there were possible adverse events. A total of 17 investigators replied, sharing individual-level patient data (IPD) from 15 studies, which represent a database of 1,088 children weighing less than 15 kg treated with oral ivermectin. Overall 18 adverse events were reported in 1.4% (15/1,088) of children, all of which were mild and self-limiting. No serious adverse events were recorded. These data suggest that ivermectin is safe for use in children weighing less than 15 kilograms. Further data from well-designed clinical trials are needed to assess the safety of oral ivermectin at escalating doses in children weighing less than 15 kg.

## Introduction

Ivermectin is a safe broad-spectrum anthelminthic drug registered for the treatment of several neglected tropical diseases (NTDs) including onchocerciasis, lymphatic filariasis, scabies, and strongyloidiasis. These NTDs frequently afflict small children but ivermectin is not currently indicated or licensed for use in children weighing less than 15 kg because of a lack of safety evidence. Because of this contraindication, alternatives to oral ivermectin are used in children weighing less than 15 kg that may be less effective (*e*.*g*. topical creams for scabies) or potentially toxic (*e*.*g*. lindane for scabies, thiabendazole for strongyloidiasis).

Ivermectin was developed initially for human use in the control of *Onchocerca volvulus* transmission because it kills microfilariae rapidly and it inhibits release of microfilariae from the gravid female worms. It is unlikely that infants and young children contribute substantially to the transmission of *O*. *volvulus* because of the biology of the parasite; it takes more than one year from entry of L3 larvae into the human body for these larvae to mature, find a mate, become fertile, and start releasing microfilariae [1,2]. Field evaluations indicate that it is very uncommon for children aged less than 5 years to be microfilaridermic [1,3]. Therefore, during drug development, it was not considered necessary to evaluate the safety and efficacy of ivermectin in children weighing less than 15 kg for inclusion in mass drug administrations (MDAs) for onchocerciasis control.

While there is a contraindication on the ivermectin package insert for treating children weighing less than 15 kg, it is likely that there have been millions of children weighing less than 15 kg who have been given ivermectin during MDA campaigns for onchocerciasis and lymphatic filariasis in Africa. Over 400 million people were treated with ivermectin during MDAs in 2019 alone, primarily in Africa, with over four billion treatments administered via MDA to date [4]. To expedite ivermectin delivery during MDAs, ivermectin dosing is usually based on height and not weight [5] with a cut-off for administration of 90 cm [4]. Based on WHO Child Growth Standards, the median weight for a 90 cm child is 12.7 kg for boys and 12.5 kg for girls, thus a 90 cm child weighing 15kg would be on the 97^th^ weight percentile for boys and the 98^th^ percentile for girls [6]. Therefore, it is likely that children with a weight between 11 and 15 kg in practice are routinely administered ivermectin during MDAs. As NTDs may cause growth stunting in children, for example infection with soil-transmitted helminths (STHs) or onchocerciasis-associated Nakalanga syndrome [7] some children do not reach 90 cm until they are older, and thus are denied ivermectin during MDA campaigns which could eliminate the very parasites causing their stunted growth. Infants one to three months of age have been treated with oral ivermectin (200 μg/kg) for scabies, with one dose [8,9], or two doses spaced one to two weeks apart [9,10], with no neurological events reported.

In mammals, ivermectin is prevented from crossing the blood brain barrier (BBB) by active efflux via P-glycoprotein (P-gp) at the luminal membrane of capillary endothelial cells [11]. However, neurotoxic complications have been observed in certain dog breeds (*e*.*g*. Collies, Sheepdogs) following ivermectin treatment. It was discovered that these dog breeds have a four base pair deletion in the *ABCB1* gene [12] that causes complete loss of transport function of P-gp allowing entry of ivermectin into the central nervous system [13]. A recent case report illustrates that nonsense mutations in the *ABCB1* gene leading to a loss of function of P-gp associated with a neurological adverse event in a 13-year-old boy following a single dose of ivermectin (200 μg/kg) [14]. Concerns have been raised regarding the use of ivermectin in infants due to a partially formed BBB.

During early drug development, preclinical toxicity studies of ivermectin were performed in neonatal rats and macaques. Neurotoxicity was observed in 1- to 2- day old rats (CRCD strain), which manifested as ataxia, ptosis, and decreased activity. The rat neonatal LD_50_ was 2.3 mg/kg, approximately twenty-fold lower than the adult LD_50_ of 42.8–52.8 mg/kg [15]. However, neonatal rats may not be an ideal model for ivermectin toxicity for human infants. In general, human and primate BBB development begins earlier in gestation and proceeds more rapidly than it does in rodents [16]. By birth, the ratio of human P-gp expression of newborns to adults is higher in humans [17] compared to rats [18]. Neonatal macaques are a better model for humans. Macaques aged 6–13 days old given oral ivermectin (100 μg/kg) for 14 days experienced no AEs [15]. In humans, P-gp reaches adult levels of expression three to six months after birth [16].

A systematic review of the safety of oral ivermectin in small children published previously [19], concluded that the limited available literature suggests that ivermectin is well tolerated by small children with no serious or long-term adverse effects. Here we provide an updated and more detailed review of the published literature on the safety of ivermectin in children weighing less than 15 kg using individual-level patient data (IPD).

## Methods

### Ethics statement

Data included in this analysis were obtained in accordance with ethical approvals from the location of origin. Data were requested to be shared anonymized. A non-human subjects use protocol was established for the acquisition of IPD, reviewed by the Walter Reed Army Institute of Research (WRAIR#2458). Consent was not obtained because data were analyzed anonymously.

### Literature review

Reports on the use of ivermectin in children weighing less than 15 kg were searched for on the following databases: MEDLINE via PubMed, Web of Science, Ovid Embase, LILACS, Cochrane Database of Systematic Reviews, TOXLINE, and ClinicalTrials.gov. Databases were searched in accordance with the Preferred Reporting Items for Systematic Reviews and Meta-Analyses of individual participant data (PRISMA) statement (S1 Checklist) [20]. Clinical trials, case series, case reports, and database entries published between 1 January 1980 to 25 October 2019 in any language describing ivermectin use in small children were identified. The US Food and Drug Administration, the Australian Adverse Drug Reactions Advisory Committee, the EudraVigilance system, and Vigibase (maintained by the WHO Collaborating Centre for International Drug Monitoring) were contacted for case reports of ivermectin adverse reactions in children weighing less than 15 kg however, these databases do not collect weights of individuals so no data could be contributed to this study. The protocol was registered in the International Prospective Register of Systematic Reviews (PROSPERO): CRD42017056515. Three independent investigators undertook the review process and extracted the data (PJ, WM, KCK), resolving discrepancy through discussion. Search terms included but were not restricted to: (soolantra OR sklice OR oramec OR mk933 OR "mk 933" OR ivomec OR eqvalan OR eqvalen OR epimer OR diapec OR cardomec OR stromectol OR mectizan OR ivermectin) AND (child or children OR newborn* OR infan* or new-born* or perinat* or neonat* or baby* or babies or toddler* or boy or boys or girl or girls or kid or kids or pediatric* or paediatric* or peadiatric* or prematur* or preterm* OR "low birth weight" or vlbw or lbw or preschool*). Screening was based on the title and abstract, and if treatment based on age (≤5 years old) or weight (<15 kg) was not clearly defined, then a review of the manuscript was performed. To confirm if ivermectin was used in children weighing less than 15 kg, authors of possible reports of ivermectin use in small children were contacted via email on at least 3 attempts in English, and when warranted in French, Portuguese, Spanish, or Swedish. In some cases, attempts were made to call and/or message authors via ResearchGate.

Lead investigators of identified studies were contacted to share individual-level patient data IPD. Only studies including weight of ivermectin-treated children less than 15 kg and adverse events data were considered for the final IPD meta-analysis. Adverse events were defined as the appearance or worsening of any undesirable sign, symptom, or medical condition after starting the study drug, even if the event was not considered related to the study drug. Serious Adverse Events (SAEs) were defined as any untoward medical occurrence that at any dose: resulted in death; was life-threatening; required inpatient hospitalization or resulted in prolongation of existing hospitalization; resulted in persistent or significant disability/incapacity; was a congenital anomaly/birth defect; or was a medically important event or reaction [21].

Additional patient-level data including disease indication for ivermectin use, manufacturer, brand name, dose, regimen, and delivery method were requested. IPD shared with the WorldWide Antimalarial Resistance Network (WWARN) were curated and standardized as described in a data management plan [22]. Any data inconsistencies or missing information were resolved directly with the investigators. Descriptive summaries were made to describe the risk of selection bias and reporting bias from the IPD contributing studies.

### Outcome

The primary outcome was the incidence of adverse events in children weighing less than 15 kg that ingested oral ivermectin.

### Statistical analysis

Since data were limited and heterogenous, mostly descriptive analyses were conducted. Incidences of AEs, their grading and causality classification, as determined by the on-site principal investigator or physician in each study were pooled and analyzed. Incidence of any AEs in children weighing less than 15 kg who were treated for scabies was estimated by pooling data from cohort, MDA or randomized studies (excluding case reports/series) using fixed logistic regression model (stata command *metapreg*). Heterogeneity between studies was quantified using I^2^ measure. Exact confidence intervals are presented for individual studies and for rate of SAE. Risk of bias was assessed based on study design and AE elicitation method.

## Results

### Systematic review summary

**Fig 1:**
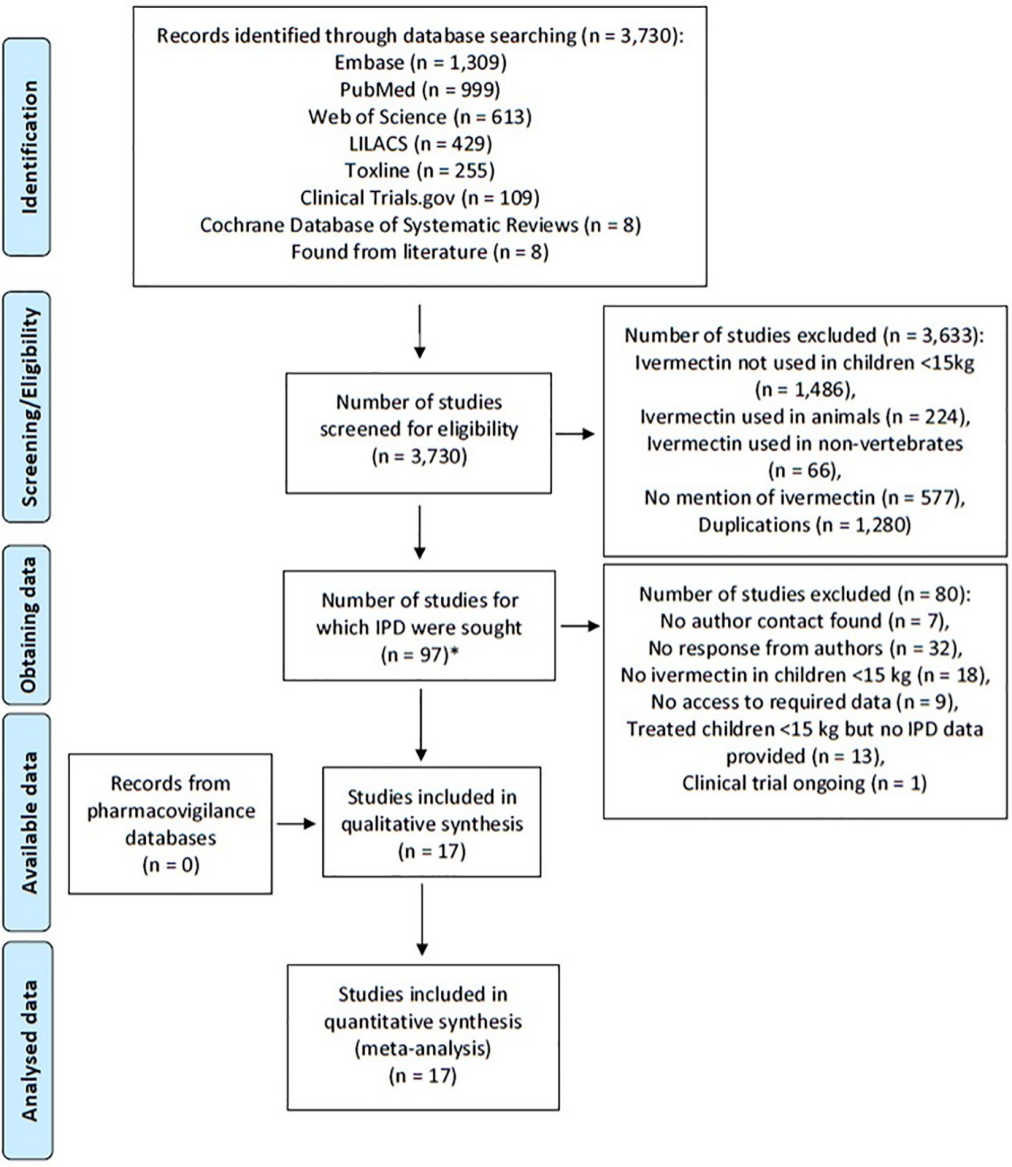
PRISMA IPD flow diagram. Fig 1 depicts the total number of studies identified from systematic review, rationale for exclusion, and number of studies included in the final analyses. * See S1 Table for list of studies and rationale for inclusion or exclusion from the review.

A total of 3,730 potential publications were identified and assessed for eligibility from literature database searches, of which 97 were selected for potential inclusion in the review (Fig 1). We attempted to contact all authors of the publications, and received responses for only 59.8% (58/97) of publications. Of responding authors, 29.3% (17/58) successfully provided IPD on ivermectin use in children weighing less than 15 kg, while 31.0% (18/58) confirmed that oral ivermectin was not provided to children weighing less than 15 kg, 8.6% (5/58) reported they no longer had access to the data, 6.9% (4/58) reported that ivermectin was used in children weighing less than 15 kg but weight or adverse event data were not recorded or were not available, 22.4% (13/58) reported they used ivermectin in children weighing less than 15 kg but failed to provide the IPD data, and 1.7% (1/58) reported that children weighing less than 15 kg will be treated with oral ivermectin but the study has not started yet (S1 Table). Overall, 17 reports describing 15 studies provided IPD. Characteristics of the 15 included studies are summarized in Table 1. Study designs utilized include: case reports 46.7% (7/15; 10 subjects), case series 26.7% (4/15; 175 subjects), cohort studies 6.7% (1/15; 4 subjects), MDA 6.7% (1/15; 838 subjects), a prospective observational study 6.7% (1/15; 17 subjects), and a randomized controlled trial 6.7% (1/15; 44 subjects). No issues were identified in IPD integrity.

**Table 1:** Study characteristics.

Study ID	Pubmed ID	Country	Study Type	Year(s)	Indication	Brand Name	Manufacturer	Number Doses	Days b/w doses	Dose μg/kg	Number of Subjects	AE Method	Cohort Description
1	29576324	France	Prospective observational study	2015–2017	Scabies	Stromectol	MSD France	Two	7–10	150–400	17	Passive reporting	*Sarcoptes scabei* positive children seen at one of seven French departments of dermatology
2	28063594 29576324	France	Case report	2017	Scabies	Stromectol	MSD France	Two	10	100	1*	Passive reporting	Single child seen at clinic
3	31073620	France	Case series	2017	Cutaneous Larva Migrans	Stromectol	Unknown	One	n/a	200	1	Observed in clinic	Autochthonous cases in France
4	30468533	France	Case report	2017	Cutaneous Larva Migrans	Stromectol	MSD France	One	n/a	272	1	Observed in clinic	Single child seen at clinic
5	23094746	Venezuela	Case report	2012	Strongyloidiasis	Kilox	Laboratorios Bussie	Two	1	200	1	Observed in clinic	Single child seen at clinic
6	30857778	France	Case report	2019	Pthiriasis	Stromectol	MSD France	Two	8	400	1	Passive reporting	Single child seen at clinic
7	16045712	Mexico	Case report	2005	Scabies	Unknown	Unknown	One	n/a	200	1	Passive reporting	Single child seen at clinic
8	no PMID**	Brazil	Case report	2002	Crusted scabies	Unknown	Unknown	Two	7	200	1	Observed in clinic	Single child seen at clinic
9	26962825	Spain	Case report	2013	Strongyloidiasis	Stromectol	MSD France	Two	1	200	4	Observed in the clinic	*Strongyloides stercoralis* positive children; one published and three unpublished case reports
10	29165211	Peru	Case series	2012–2015	Myiasis	Quanox oral	Siegfried	One	n/a	300	1	Observed in the clinic	Single child seen at clinic
11	12139665	Mexico	Case series	2002	Crusted scabies / Scabies/ Cutaneous Larva Migrans	Unknown	Unknown	One or two	14	150–200	4	Passive reporting	*Sarcoptes scabei* or cutaneous larva migrans positive children at least 14 mo that were not able to be treated with topical creams
12	31344258	France	Case series	2012–2015	Scabies	Stromectol	MSD France	One or two	7–15	94–556	169	Reported at follow up visit at 14 days	*Sarcoptes scabei* positive children weighing less than 15 kg; standardized anonymous form was sent to members of the French Society of Paediatric Dermatology
13	30223985	Solomon Islands	MDA trial	2015	Scabies MDA	Stromectol	MSD Australia	One or two	3–19	200–400	838	Reported at second visit	All residents eligible to participate, excludes pregnant and breastfeeding women or children weighing less than 12.5kg
14	29617737	Côte d’Ivoire	Randomized Controlled Trial	2017	Trichuriasis	milled 0.5 mg tablet	Elea Laboratorios	Single dose	n/a	81–500	44	Reported at follow up visits at 3,24,72 hours	*Trichuris trichiura* positive children 2–12 yo
15	27548286	Cambodia	Cohort study	2012–2014	Strongyloidiasis	Stromectol	MSD Netherlands	Single dose	n/a	200	4	Passive reporting	*Strongyloides stercoralis* positive residents over 2 yo
16	29059195 27548286	Cambodia	Cohort study	2012–2014	Strongyloidiasis	Stromectol	MSD Netherlands	Single dose	n/a	200	4***	Passive reporting	*Strongyloides stercoralis* positive residents over 2 yo
17	28070007	Guadeloupe	Case report	2013	Parasitic disease of unknown origin before the diagnosis of angiostrongyliasis	Stromectol	MSD France	Single dose	n/a	353	1	Observed in the clinic	Single child seen at hospital

* Single case included in Study ID 1

** No Pubmed ID; Dermatol. Argent; 8(3):136–140, jul.-aug. 2002. Available at: https://www.dermatolarg.org.ar/index.php/dermatolarg/article/view/357/168

*** These four cases included in Study ID 15

### IPD summary

From the 17 reports, there were 1,088 confirmed instances in which oral ivermectin was given to children weighing less than 15 kg. The majority of the treated children (80.2%, 867/1,081) were younger than five years of age with an overall median age of 36 months (range 1–132 months). Median weight was 13.0 kg (range 4.0–14.9 kg). Height was only available in 5 out of 15 studies, for 10.9% (119/1,088) of children, with a median of 84 cm (range 54–103 cm) and 28.6% (34/119) were 90 cm or taller.

**Fig 2:**
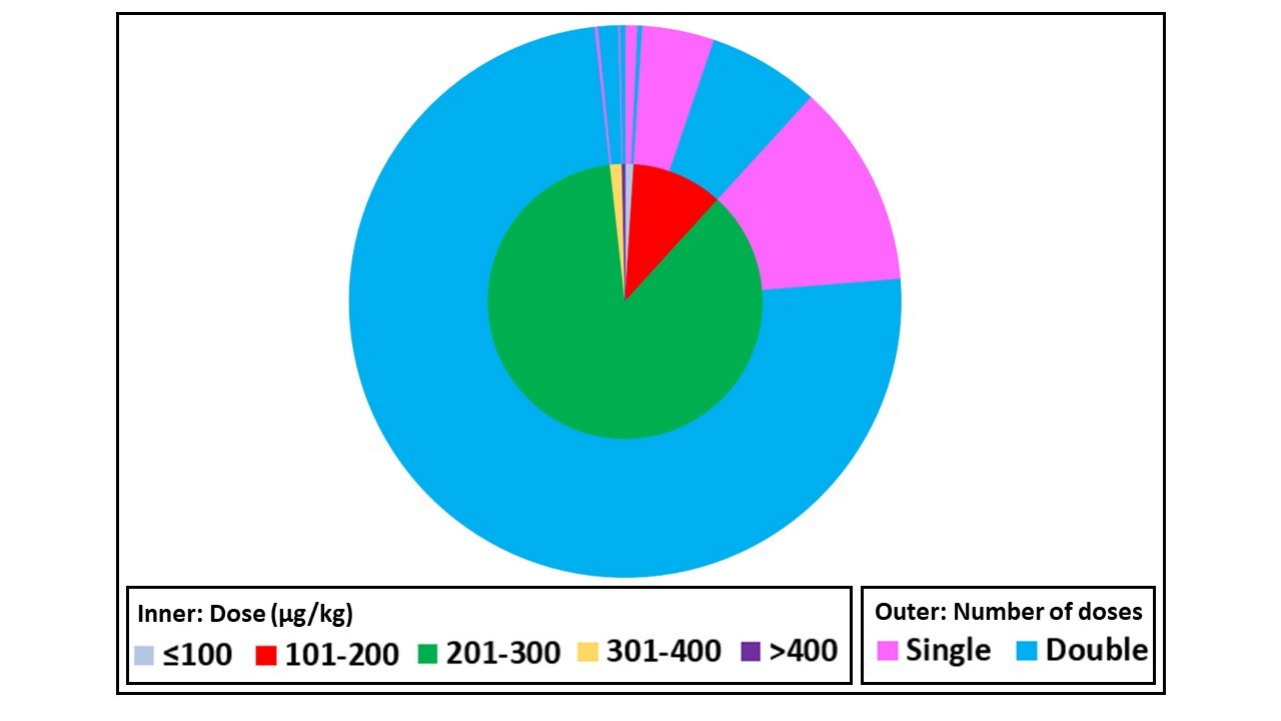
Ivermectin dose and number administered to children weighing less than 15 kg. Fig 2 depicts the ivermectin dose administered in μg/kg (inner ring) and the number of doses administered (outer ring) that were collected for the IPD database (n = 1,088).

Of ivermectin-treated children, 82.8% (901/1,088) received two doses of ivermectin anywhere from 1 to 19 days apart. Although 200 μg/kg of ivermectin is the standard oral dose for most NTDs, 86.5% (941/1,088) children received a dose between 201–300 μg/kg and 1.7% (19/1,088) received doses above 300 μg/kg (Fig 2). Overall, the median dose administered was 221 μg/kg (range 81–556 μg/kg). The doses administered were significantly higher in the two-dose regimen (median 231 μg/kg, range 94–556, n = 901) compared to the single-dose regimen (median 214 μg/kg, range 81–500 μg/kg, n = 187), (p<0.001, Mann Whitney test).

Oral ivermectin was administered to children weighing less than 15 kg for a total of nine indications including: scabies MDA 77.0% (838/1,088), scabies individual treatment 17.3% (188/1,088), trichuriasis 4.0% (44/1,088), strongyloidiasis 0.8% (9/1,088), cutaneous larva migrans 0.4% (4/1,088), crusted scabies 0.2% (2/1,088), myiasis 0.1% (1/1,088), pthiriasis 0.1% (1/1,088), and parasitic disease of unknown origin 0.1% (1/1,088). Instances of ivermectin administration to children weighing less than 15 kg were reported from ten countries including: Solomon Islands 77.0% (838/1,088), France 17.4% (189/1,088), Côte d’Ivoire 4.0% (44/1,088), Mexico 0.5% (5/1,088), Cambodia 0.4% (4/1,088), Spain 0.4% (4/1,088), Brazil 0.1% (1/1,088), Guadeloupe 0.1% (1/1,088), Peru 0.1% (1/1,088), and Venezuela 0.1% (1/1,088).

In total, 18 AEs were reported from 1.4% (15/1,088) of ivermectin-treated children and none of the AEs (0/18, 95%CI 0–0.33%) were deemed SAEs (Table 2). The most common AEs reported were diarrhea 0.4% (4/1,088) and eczema 0.5% (5/1,088). Headache, itching and vomiting were each reported twice 0.2% (2/1,088) and joint pain, abdominal pain, and symmetrical edema of the feet were each reported once 0.1% (1/1,088) (Tables 2 and S2). Twelve patients reported only one AE, while three patients reported 2 AEs: headache and diarrhea (n = 1), abdominal pain and vomiting (n = 1), diarrhea and itching (n = 1). All AEs were deemed possibly related to ivermectin administration, except for the joint pain which was classified as unlikely related to ivermectin administration. Adverse events were related to scabies treatment or MDA for scabies in 50% (9/18), trichuriasis in 44.4% (8/18), and strongyloidiasis in 5.6% (1/18) of patients. A pooled prevalence of AEs in the scabies, crusted scabies, or scabies MDA data derived from 3 studies, was estimated as 0.88% (95%CI 0.48–1.68) (Table 3). Seven of the AEs occurred in children that were co-administered either topical creams for scabies (4/7), oral azithromycin (2/7), or intravenous iron plus vitamin supplements (1/7) (Table 2). No adverse events were reported in the 1.3% (14/1,081) of children three months or younger. Of 128 children who received ≤200 μg/kg ivermectin, 7.0% (9/128) experienced an AE, of 960 children who received >200 μg/kg ivermectin 0.6% (6/960) reported an AE, and of 19 children who received >300 μg/kg ivermectin none reported an AE. All AEs were considered mild, self-limiting, and resolved without further intervention.

**Table 2:** Listing of individual adverse events (n = 18) following ivermectin ingestion. None of the AEs were classified as serious adverse events.

AE ID	Patient ID	Study ID	AE term	Relation to IVM	Time Reported (Days)	Weight (kg)	Age (mo)	Number of doses	Dose (μg/kg)	Indication	Concomitant Medication
1	1	11	Diarrhea	Possible	10	10	10	2	300	Scabies	Topical Cream
2	2	13	Diarrhea	Possible	1	10	24	1	100	Trichuriasis	
3	3	13	Diarrhea	Possible	3	11	24	1	182	Trichuriasis	
4	4	12	Diarrhea	Possible	8	13	24	2	231	Scabies MDA	Azithromycin
5	5	11	Eczema	Possible	10	10	12	2	150	Scabies	
6	6	11	Eczema	Possible	7	10	16	2	200	Scabies	Topical Cream
7	7	11	Eczema	Possible	10	10	18	2	150	Scabies	Topical Cream
8	8	11	Eczema	Possible	8	10.1	14	2	297	Scabies	Topical Cream
9	9	11	Eczema	Possible	8	11	31	2	205	Scabies	
10	2	13	Headache	Possible	1	10	24	1	100	Trichuriasis	
11	10	13	Headache	Possible	1	13	60	1	192	Trichuriasis	
12	3	13	Itching	Possible	3	11	24	1	182	Trichuriasis	
13	11	13	Itching	Possible	1	13.2	24	1	114	Trichuriasis	
14	12	12	Joint Pain	Unlikely	8	13	36	2	231	Scabies MDA	Azithromycin
15	13	13	Abdominal Pain	Possible	1	14	60	1	107	Trichuriasis	
16	14	11	Vomiting	Possible	10	11	32	2	273	Scabies	
17	13	13	Vomiting	Possible	3	14	60	1	107	Trichuriasis	
18	15	4	Symmetrical Edema of the Feet	Possible	1	4.7	18	2	200	Strongyloidiasis	IV Iron and Vitamin Supplement

AE, adverse event; IVM, ivermectin; kg, kilograms; mo, months; IV, intravenous

**Table 3:** Pooled AE incidence in children with Scabies, Crusted scabies, or participating in MDAs for scabies (excluding case studies) from a fixed effects logistic regression model. I^2^ statistics (variation in log-odds attributable to heterogeneity) = 21.4%.

Study ID	Number of patients	Number of AE	AE incidence (%)	95% CI
1	17	0	0.00%	0.00% - 19.51%
12	169	7	4.14%	1.68% - 8.35%
13	838	2	0.24%	0.03% - 0.86%
**Overall—Fixed Effects**			**0.88%**	**0.46% - 1.68%**
Bécourt et al. 2013	15	2	13.33	1.66–40.46
**Overall—Fixed Effects**			**1.06%**	**0.59% - 1.90%**

### Risk of bias

A single patient case report or small case series does not allow for estimation of AE frequency, but provides descriptive or narrative results. Therefore, case reports or case series in our review (study IDs 2–11,17) [8,23–32] with patients (n = 16) seen in the clinic were categorized as high risk of selection and reporting bias.

Among the remaining studies, low risk of selection bias is expected in the randomized control study (study ID 14) [33] (n = 44) and the MDA trial (study ID 13) [34] in which all eligible persons in the study area were treated (n = 838) and participants from randomly selected villages were followed up for AEs, and moderate risk in the prospective studies (study IDs 1,15,16) [35–37] (n = 21). Only two studies actively asked for AEs (study ID 12,14) [9,33] (n = 213), while MDA (study ID 13) [34] (n = 838) and cohort studies (study ID 15,16) [35,36] (n = 4) used passive reporting of AEs, and were categorized a high risk for reporting bias.

One study identified by the review [10] could not be included in the analysis because AEs were not linked to individual patients. The article reported that among 15 children weighing<15kg treated for scabies, two experienced AEs (one was nervous and irritable and the other scratched intensely). Including this study would slightly increase the pooled AE frequency to 1.06% (95% CI 0.59–1.90%) (Table 3). In any other studies (n = 79) for which IPD could not be obtained, data published could not be used in the synthesis as the AEs and number of patients studied were not presented explicitly in the weight group of interest. Consequently, we cannot assess representativeness of the IPD collated in relation to all ivermectin use data available in this weight group.

## Discussion

This systematic review identified 97 reports where ivermectin was potentially used in children weighing less than 15 kg for numerous indications including: scabies MDA, scabies, crusted scabies, strongyloidiasis, onchocerciasis, lymphatic filariasis, baylisascariasis, head lice, pubic lice, cutaneous larva migrans, gnathostomiasis, trichuriasis, tungiasis, trombidiasis, rosacea, capillariasis, myiasis, intestinal acariasis, and parasitic disease of unknown origin. However, IPD reporting both the weight and adverse events could only be acquired from 17 reports, accounting for 1,088 children, treating the following indications: scabies MDA, scabies, crusted scabies, strongyloidiasis, pthiriasis, cutaneous larva migrans, trichuriasis, myiasis, and parasitic disease of unknown origin (Table 1). The evidence summarized here indicates that oral ivermectin caused AEs in 1.4% (15/1,088) children weighing less than 15 kg and there were no SAEs reported. With a sample size of 1000, the probability of observing at least one AE with prevalence of 1 in 1000 is 0.632, and >0.999 for adverse events with incidence 1 in 100. Thus, the upper 95% confidence interval for the true incidence of AEs following ivermectin administration to children <15kg is 1 in 362.

All data contributors rated the AEs as being possibly caused by ivermectin treatment with the exception of joint pain which was rated as unlikely to be caused by ivermectin. Of the reported AEs, headache 2 (0.18%), vomiting 2 (0.18%), abdominal pain 1 (0.09%), diarrhea 4 (0.37%), and itching 2 (0.18%) have all previously been described for ivermectin and in the current study occurred at rates less than stated on ivermectin package inserts [38,39]. Our study results therefore suggest that ivermectin is as safe and well-tolerated in children weighing less than 15 kg as indeed it is for persons weighing more than 15 kg. One patient given oral ivermectin for strongyloidiasis had symmetrical edema of the feet, but this could not be directly attributed to ivermectin because the child was extremely malnourished and was also given intravenous fluids, iron, and supplements [26]. A limitation of this study is the lack of responses from several authors 32.0% (31/97) and inability to contribute IPD-level data after confirmation of oral ivermectin use in children weighing less than 15 kg 13.4% (13/97) from published reports.

This evidence has been compiled into an IPD database, held at WWARN, and available upon request to by contacting the study authors. Our hope is that this database can be used for regulatory review regarding the safety of ivermectin in children weighing less than 15 kg. These data can be difficult to compile as they are generated from a wide range of treatment indications from around the world. The following sections discuss key issues surrounding use of ivermectin in children weighing less than 15 kg.

### Ivermectin treatment and mass drug administration for scabies

Several trials of ivermectin MDA for the control of scabies have been performed in the South Pacific [34,40,41]. Adequate population coverage is critical for the success of scabies MDA. The use of oral ivermectin has logistical and compliance advantages over topical creams. Furthermore, MDA of oral ivermectin was found to be more effective than topical permethrin [40]. Children not treated by ivermectin during MDA serve as a reservoir for future community re-infection unless adequately treated with topical treatments. One MDA trial for scabies in the Solomon Islands co-administered ivermectin and azithromycin for the control of scabies and impetigo. Azithromycin is indicated for use in children weighing 12.5 kg or more, and in the trial in the Solomon Islands the 12.5 kg limit was also used for ivermectin for pragmatic reasons. A total of 838 children weighing 12.5 to less than 15 kg received ivermectin as part of MDA, and only two adverse events were reported in this group [34].

In 2018, Ethiopia performed the largest scabies ivermectin MDA to date, treating 1,634,271 persons with oral ivermectin. Drug administration was not based on height or weight but by age. Children two to six years old were administered a single 3 mg ivermectin tablet, with 147,380 children in this age range treated [42]. Based on WHO Child Growth Standards, the median weight for a 24-month-old child is 12.2 kg for boys and 11.5 kg for girls, with children achieving a median 15.0 kg weight at 40 months for boys and at 42 months for girls [6]. Enbiale et al. report that roughly 30% of children aged two to six years old were given more costly and logistically difficult to administer topical permethrin or sulfur creams instead of oral ivermectin, which may have occurred because of confusion with onchocerciasis guidelines indicating that children under five years of age should not be treated with ivermectin [42]. In this review, of the children weighing less than 15 kg treated with oral ivermectin, 19.8% (214/1,081) were five years of age or older. While this data set is limited, it suggests that treating by age with five years old as a cutoff would still frequently provide ivermectin to children weighing less than 15 kg. Of the children weighing less than 15 kg treated with oral ivermectin, 28.6% (34/119) were 90 cm or taller. This illustrates that the 90 cm cutoff for ivermectin administration during MDAs means that children weighing less than 15 kg are routinely treated with ivermectin. Establishing the safety of ivermectin in children weighing less than 15 kg would remove the imprecise weight, height, and age restriction barriers and facilitate more streamlined ivermectin MDAs.

Scabies is recognized in France as a common clinical problem, including in small children. In 2014, the French Medicines Regulatory Agency “Agence Nationale de Sécurité du Médicament”, published a decision algorithm for treatment of scabies in children aged less than one year. This algorithm recommends treatment with two doses of oral ivermectin (200 μg/kg) one week apart in infants who fail scabies treatment with permethrin or benzyl benzoate topical creams, or have a contraindication for topical esdepallethrin spray (*e*.*g*. asthma) [43]. A survey of French dermatologists found that oral ivermectin was unlikely to be used in infants with common scabies weighing less than 10 kg (15%). However, willingness to use oral ivermectin in children weighing less than 10 kg increased: if the child had asthma (25%), after topical cream failure (40%), complications with impetigo (60%), profuse scabies (70%), or if the child was unlikely to be seen again follow-up (65%). Furthermore, survey respondents were willing to treat scabies in children less than three months with oral ivermectin [44]. Indeed, in this current review, of the 14 children that were three months or younger that were administered oral ivermectin, all of them were treated for scabies and 92.9% (13/14) were treated in France. France appears to be the first country with clear guidance to use oral ivermectin off-label for treatment of scabies in children weighing less than 15 kg.

### Ivermectin treatment for soil-transmitted helminthiasis and strongyloidiasis

Ivermectin has been used for the treatment of young children weighing less than 15 kg with infections due to STHs or *Strongyloides stercoralis*. The combination of ivermectin and albendazole is superior to either drug alone for the treatment of *Trichuris trichiura* [45] and albendazole is superior to ivermectin for the treatment of hookworm. The Global Program for the Elimination of Lymphatic Filariasis (GPELF) co-administered ivermectin and albendazole to 52 million school-aged children via MDA in 2015 [46] and this combination has been added to the WHO List of Essential Medicines for Children [47]. However, the combination is still not used in children weighing less than 15 kg because of the ivermectin contraindication. Part of the GPELF rationale for albendazole and ivermectin co-administration was increased secondary effects against STHs [48]. The triple-drug therapy of ivermectin, diethylcarbamazine, and albendazole (IDA) was recently shown to be superior for the treatment of lymphatic filariasis [49] and Merck has pledged an additional 100 million ivermectin treatments annually to use in triple-drug therapy IDA MDAs outside of Africa [50]. However, with the success of the GPELF against lymphatic filariasis, MDA is now scaling back in many areas. If community-wide MDA programs with ivermectin and albendazole are to continue in the future, then STH programs will have to shift strategies from school-based administration of albendazole to community-wide MDAs with combinations of ivermectin and albendazole, and inclusion of children weighing less than 15 kilograms could be considered.

A recent clinical trial demonstrated that ivermectin for *T*. *trichiura* infection is safe in preschool-aged children, some of whom weighed less than 15 kg (n = 44) [33], but safety studies of the combination of ivermectin and albendazole in children weighing less than 15 kg are lacking. STH infection at a young age is associated with growth stunting, impairment of cognitive development, and reduced school attendance. Co-administration of ivermectin and albendazole during MDAs to small children infected with STHs could positively impact childhood development in many settings.

Expanding ivermectin use for children weighing less than 15 kg during MDAs could have marked health benefits for this group through impact on *S*. *stercoralis* burden. Repeated annual ivermectin MDA for onchocerciasis control in Colombia [51] and Ecuador [52] demonstrated marked and sustained reductions in *S*. *stercoralis* prevalence. Three rounds of annual ivermectin and albendazole MDA in Argentina [53] and two rounds of annual ivermectin MDA in Australia [54] led to marked reduction on *S*. *stercoralis* prevalence. Strongyloidiasis can be life threatening in small children and ivermectin is a safe and effective cure for infected children weighing less than 15 kg [26,29,35,36,55].

### Ivermectin for myiasis

The administration of oral ivermectin for myiasis has proven to be effective as it prevents complications in surgery and reduces the difficulty of mechanical removal of the larvae [56]. Children are vulnerable to nasal [57], oral [57,58], ocular [59–61], and auricular myiasis [57,62]. Treatment of children with oral ivermectin (200–350 μg/kg) for myiasis is effective [30,59–61,63] but use in children weighing less than 15 kg has been extremely limited due to the current contraindication [38,39].

### Onchocerciasis, onchocerciasis-associated epilepsy and Nodding Syndrome

While the prevalence of onchocerciasis microfilaridermia in children aged less than five years is expected to be low, these children can become infected in areas of high *O*. *volvulus* transmission and present disease manifestations, especially pruritus [64]. The presence of troublesome itching may produce general fatigue, insomnia, and distraction at school [65,66]. It is possible that itching could be exacerbated in *O*. *volvulus* infected children weighing less than 15 kg that were treated with ivermectin, however, no studies were identified that assessed ivermectin in this weight class and disease combination. Trials in *O*. *volvulus* infected children weighing less than 15 kg are recommended before large scale ivermectin MDA rollout in onchocerciasis endemic regions in this weight class. In addition, the relative risk of excess mortality associated with onchocerciasis is higher, for a given microfilarial load, for younger age groups [67]. Furthermore, there is a consistent association between onchocerciasis and the incidence of onchocerciasis-associated epilepsy and Nodding Syndrome when the infection is intense and occurs at a very young age [68–73]. Nodding Syndrome, a form of epilepsy, may be caused by an autoimmune reaction to *O*. *volvulus* infection [74] and afflicts children between three and 18 years old [75]. Onchocerciasis-endemic regions where ivermectin MDAs are routinely performed have reduced [76–79]. All of these observations suggest that inclusion of children weighing less than 15 kg in ivermectin MDA will benefit child health in onchocerciasis endemic areas.

### Ivermectin use for malaria control

Ivermectin has been proposed as a novel malaria control tool due to its ability to kill *Anopheles* mosquitoes and ivermectin MDA can suppress *Plasmodium* transmission [80–82]. When using ivermectin MDA as a malaria control agent, coverage is the most critical component of efficacy [83]. The contraindication of ivermectin for children weighing less than 15 kg excludes roughly 20% of the population in malaria endemic areas, potentially inhibiting MDA efficacy. In Africa, children under five years old are frequently bitten by *Anopheles* as evidenced by high rates of new malaria infections in this population [82]. Untreated small children serve an important reservoir of *Plasmodium* and can facilitate onwards transmission to mosquitoes.

Clinical trials have evaluated the safety, and efficacy of ivermectin along with the antimalarial drugs artemether-lumefantrine [84] and dihydroartemisinin-piperaquine [85,86] in adults. However, safety data in children and infants are needed in order to expand administration of these combinations to younger age groups. Currently in the African Sahel, seasonal malaria chemoprophylaxis (SMC) with sulfadoxine-pyrimethamine plus amodiaquine (SP-AQ) is given annually to 10–15 million children less than five years old, as an effective tool to reduce clinical incidence of severe malaria. Combining ivermectin MDAs with SMC would simultaneously target the parasite and vector, likely further reducing malaria in young children [87]. Before ivermectin and SP-AQ can be co-administered, safety and efficacy studies should be performed. Finally, ivermectin has been shown to inhibit liver-stage development of *Plasmodium berghei* [88], but its prophylactic efficacy in humans is debatable. Field evidence of repeated ivermectin (150 μg/kg) MDAs demonstrated that ivermectin treatment reduced the likelihood of infection and number of *P*. *falciparum* clones per infection beyond predicted community-wide transmission benefit, suggestive of ivermectin liver-stage prophylactic effect [82]. However, a recent controlled human malaria infection trial with single dose ivermectin (400 μg/kg) in adults failed to prevent *P*. *falciparum* infection [89]. If ivermectin can indeed inhibit *Plasmodium* development in treated humans, then this makes the inclusion of children weighing less than 15 kg in ivermectin MDAs for malaria control even more critical as they suffer a higher burden of morbidity and mortality from malaria.

### Pediatric ivermectin formulations and dosage

Oral Ivermectin is typically delivered as 3 mg or 6 mg tablets, and while the 3 mg tablet size is relatively small, 5.5 mm diameter for Stromectol (Merck & Co., Whitehouse Station, NJ, USA), it can be difficult for young children to swallow oral tablets. Since there is no pediatric ivermectin formulation available in France, the French Medicines Regulatory Agency recommends to crush a whole 3 mg tablet for children weighing 10–15 kg or half of a three mg tablet for children weighing less than 10 kg, mix thoroughly in 10 mL of water, and administer [43]. In the clinical setting, there was no evidence of children weighing less than 15 kg choking when swallowing crushed ivermectin (3 mg) mixed in liquid (n = 169) [9]. A recent study milled three mg tablets into smaller 0.5 mg minitablets to provide to children aged two to 12 years old, many of whom weighed less than 15 kg (n = 43) [33]. In Latin America, a liquid oral formulation has been manufactured and used in several trials for treatment of young children with head lice in Colombia [90–92], and specifically in infants weighing less than 15kg for strongyloidiasis in Venezuela [26] and myiasis in Peru [30]. A liquid oral, minitablet, oro-dispersible minitablet, or multiparticulate formulation for small children would be advantageous for use in clinical treatment and MDA settings. The considerations outlined in this manuscript underline the need for the development of an adapted pediatric formulation of ivermectin.

The ideal dose of ivermectin for different indications in small children is not known. Available data suggest that adult based dosing is not appropriate for children and that dose escalation efficacy trials in children weighing less than 15 kg are needed. Recent pharmacokinetic analyses indicate that ivermectin treated children aged less than 12 years reach half the peak concentration and total exposure of adults [93] and a dose increase for young children was suggested [94]. A multi-center analysis found that ivermectin was significantly more effective at treating scabies in children weighing less than 15 kg when the dose was more than 200 μg/kg compared to less than 200 μg/kg [9]. This suggests that the standard 200 μg/kg dose of ivermectin may not reach effective levels in small children for all NTDs. This review identified 939 children treated with 201–300 μg/kg, 17 children treated with 301–400 μg/kg, and four children treated with >400 μg/kg. This limited evidence suggests that ivermectin doses greater >200 μg/kg are potentially safe and well tolerated in children weighing less than 15 kg. One limitation of this review is that most of the evidence 92.6% (1,007/1,088) is derived from two studies, one MDA study in the Solomon Islands [34] and a case series from France [9]. A multi-country, randomized, double-blind, placebo-controlled, dose escalation (200, 400, 800 μg/kg) study, dubbed the Ivermectin Safety in Small Children (ISSC) trial (NCT04332068) is currently underway, which is designed to assess the safety, pharmacokinetics, and efficacy of oral ivermectin in scabies-infected children weighing less than 15 kg [95]. An additional concern to ivermectin dosing in children weighing less than 15 kg are potential drug-drug interactions with other drugs frequently used in MDA populations (*e*.*g*. antiretrovirals) that may alter safety or pharmacokinetics.

### A call for clarity in data reporting

Age is not a limiting criterion listed on most ivermectin package inserts but weight <15 kg and/or height <90 cm when used in MDAs are directly contraindicated [38]. However, there is a common misconception reported in the literature that children aged less than five years should not be administered ivermectin. Not treating children under five years of age would further restrict ivermectin use, as these children frequently weigh 15 kg or more or are 90 cm or taller [6]. Reports of ivermectin use in case studies, series, or trials frequently group subjects by age rather than by weight, providing no conclusive evidence for ivermectin safety in children weighing less than 15 kg. Of the 97 possible reports identified in this review, only four stated the number of children weighing less than 15 kg that were administered oral ivermectin [9,10,34,95]. Here we call for greater clarity when reporting ivermectin use in children to include the weight of any child below 15 kg and reporting of any associated AEs to the individual-patient level. An IPD call for ivermectin safety in children weighing less than 15 kg has been organized by WWARN and data from future publications or clarifications to publications cited in S1 Table can be contributed by contacting the study authors.

## Conclusion

Millions of children weighing less than 15 kg are currently denied access to ivermectin treatment due to the current label indication. In order to remove this barrier and improve treatment equity, further evidence in children weighing less than 15 kg must be gathered and clearly compiled for review. The theoretical concern regarding the potential for neurotoxicity of ivermectin in infancy has not been confirmed. This IPD meta-analysis provide new evidence to be considered by medicines regulatory authorities and policy makers regarding the safety of ivermectin in children weighing less than 15 kg. The data provides limited but encouraging evidence that ivermectin is safe and well-tolerated in small children weighing less than 15 kg. The ivermectin tolerability and safety profile in children weighing less than 15 kg is similar to that in heavier individuals. Further investigation is warranted through well-designed clinical trials in children weighing less than 15 kg with the objective of optimizing dosing and characterizing the safety profile the prescribing restriction in young children can be lifted.

## Supporting information

S1 Checklist PRISMA-IPD checklist of items to include when reporting a systematic review and meta-analysis of individual participant data. Preferred Reporting Items for Systematic Reviews and Meta-Analyses–Individual Patient Data (PRISMA-IPD).
(DOCX)

S1 Table Inclusion Summary of Studies Contacted for Individual Patient-level Data. Response results and inclusion decisions of selected studies contacted by study team.
(DOCX)

S2 Table Summary of adverse events reported by study. Number and classification of adverse events reported for each study included in the systematic analysis.
(DOCX)
