# Correction: A systematic review and an individual patient data meta-analysis of ivermectin use in children weighing less than fifteen kilograms: Is it time to reconsider the current contraindication?

**DOI:** 10.1371/journal.pntd.0011053

**Published:** 2023-01-06

**Authors:** Podjanee Jittamala, Wuelton Monteiro, Menno R. Smit, Belen Pedrique, Sabine Specht, Carlos J. Chaccour, Céline Dard, Pascal Del Giudice, Virak Khieu, Annabel Maruani, Virgilio E. Failoc-Rojas, Marimar Sáez-de-Ocariz, Antoni Soriano-Arandes, Jaime Piquero-Casals, Anne Faisant, Marie-Pierre Brenier-Pinchart, David Wimmersberger, Jean T. Coulibaly, Jennifer Keiser, Franck Boralevi, Oliver Sokana, Michael Marks, Daniel Engelman, Lucia Romani, Andrew C. Steer, Lorenz von Seidlein, Nicholas J. White, Eli Harriss, Kasia Stepniewska, Georgina S. Humphreys, Kalynn Kennon, Philippe J. Guerin, Kevin C. Kobylinski

In Table 2, the Study IDs are all off by a factor of one. Please see the correct Table 2 here.

**Table 2:** Listing of individual adverse events (n = 18) following ivermectin ingestion. None of the AEs were classified as serious adverse events.

AE ID	Patient ID	Study ID	AE term	Relation to IVM	Time Reported (Days)	Weight (kg)	Age (mo)	Number of doses	Dose (μg/kg)	Indication	Concomitant Medication
1	1	12	Diarrhea	Possible	10	10	10	2	300	Scabies	Topical Cream
2	2	14	Diarrhea	Possible	1	10	24	1	100	Trichuriasis	
3	3	14	Diarrhea	Possible	3	11	24	1	182	Trichuriasis	
4	4	13	Diarrhea	Possible	8	13	24	2	231	Scabies MDA	Azithromycin
5	5	12	Eczema	Possible	10	10	12	2	150	Scabies	
6	6	12	Eczema	Possible	7	10	16	2	200	Scabies	Topical Cream
7	7	12	Eczema	Possible	10	10	18	2	150	Scabies	Topical Cream
8	8	12	Eczema	Possible	8	10.1	14	2	297	Scabies	Topical Cream
9	9	12	Eczema	Possible	8	11	31	2	205	Scabies	
10	2	14	Headache	Possible	1	10	24	1	100	Trichuriasis	
11	10	14	Headache	Possible	1	13	60	1	192	Trichuriasis	
12	3	14	Itching	Possible	3	11	24	1	182	Trichuriasis	
13	11	14	Itching	Possible	1	13.2	24	1	114	Trichuriasis	
14	12	13	Joint Pain	Unlikely	8	13	36	2	231	Scabies MDA	Azithromycin
15	13	14	Abdominal Pain	Possible	1	14	60	1	107	Trichuriasis	
16	14	12	Vomiting	Possible	10	11	32	2	273	Scabies	
17	13	14	Vomiting	Possible	3	14	60	1	107	Trichuriasis	
18	15	5	Symmetrical Edema of the Feet	Possible	1	4.7	18	2	200	Strongyloidiasis	IV Iron and Vitamin Supplement
