# Supplementary material for: A systematic review and an individual patient data meta-analysis of ivermectin use in children weighing less than fifteen kilograms: Is it time to reconsider the current contraindication?
Source: PLoS Negl Trop Dis. 2021 Mar 17;15(3):e0009144. doi: 10.1371/journal.pntd.0009144 (PMC7968658; doi:10.1371/journal.pntd.0009144)
Supplement: S1 Table — Response results and inclusion decisions of selected studies contacted by study team. (DOCX) [file pntd.0009144.s002.docx]

**S1 Table. Inclusion Summary of Studies Contacted for Individual Patient-level Data**

| **IPD Obtained** | **Reason** | **Number of Studies** | **Publications** |
| --- | --- | --- | --- |
| Yes | Children weighing less than 15 kg treated with ivermectin, provided IPD | 17 | [1-17] |
| No | No author contact could be found | 7 | [18-24] |
| No | No response from authors | 31 | [25-55] |
| No | Author deceased | 1 | [56] |
| No | Confirmed did not treat children weighing less than 15 kg with ivermectin | 18 | [57-74] |
| No | Confirmed did not have access to the data so could not verify if children weighing less than 15 kg were treated with ivermectin | 5 | [75-79] |
| No | Confirmed treated children weighing less than 15 kg with ivermectin but either did not record weight, adverse events, or did not have access to the data | 4 | [80-83] |
| No | Confirmed treated children weighing less than 15 kg with ivermectin but did not provide the data | 13 | [84-96] |
| No | Confirmed will treat children weighing less than 15 kg with ivermectin but study has not enrolled subjects yet | 1 | [97] |

**References**

1. Chaccour C, Del Pozo J. Case 23-2012: A man with abdominal pain and weight loss. N Engl J Med. 2012;367(17):1670-1.

2. Chinazzo M, Desoubeaux G, Leducq S, Bessis D, Droitcourt C, Mahe E, et al. Prevalence of Nail Scabies: A French Prospective Multicenter Study. J Pediatr. 2018;197:154-7.

3. Dard C, Piloquet J-E, Qvarnstrom Y, Fox L, M'Kada H, Hebert J-C, et al. First Evidence of Angiostrongyliasis Caused by *Angiostrongylus cantonensis* in Guadeloupe, Lesser Antilles. Am J Trop Med Hyg. 2017;96(3):692-7.

4. Del Giudice P, Hakimi S, Vandenbos F, Magana C, Hubiche T. Autochthonous Cutaneous Larva Migrans in France and Europe. Acta Derm Venereol. 2019;99(9):805-8.

5. Failoc-Rojas V, Molina-Ayasta C, Salazar-Zuloeta J, Samamé A, Silva-Díaz H. Case Report: Myiasis due to *Cochliomyia hominivorax* and *Dermatobia hominis*: Clinical and Pathological Differences between Two Species in Northern Peru. Am J Trop Med Hyg. 2018;98(1):150-3.

6. Finon A, Desoubeaux G, Nadal M, Georgescou G, Baran R, Maruani A. Scabies of the nail unit in an infant. Ann Dermatol Venereol. 2017;144(5):356-61.

7. Forrer A, Khieu V, Schär F, Hattendorf J, Marti H, Neumayr A, et al. *Strongyloides stercoralis* is associated with significant morbidity in rural Cambodia, including stunting in children. PLoS Negl Trop Dis. 2017;11(10):e0005685.

8. Forrer A, Khieu V, Schindler C, Schär F, Marti H, Char M, et al. Ivermectin Treatment and Sanitation Effectively Reduce *Strongyloides stercoralis* Infection Risk in Rural Communities in Cambodia. PLoS Negl Trop Dis. 2016;10(8):e0004909.

9. Levy M, Martin L, Bursztejn A-C, Chiaverini C, Miquel J, Mahé E, et al. Ivermectin safety in infants and children under 15 kg treated for scabies: a multicentric observational study. Br J Dermatol. 2020;182(4):1003-6.

10. Mauleón-Fernández C, Sáez-de-Ocariz M, Rodríguez-Jurado R, Durán-McKinster C, Orozco-Covarrubias L, Ruiz-Maldonado R. Nodular scabies mimicking urticaria pigmentosa in an infant. Clin Exp Dermatol. 2005;30(5):595-6.

11. Ouedraogo M, Ventéjou S, Leducq S, Desoubeaux G, Maruani A. Crusts on the Eyelashes. J Pediatr. 2019;209(254):e1.

12. Piquero-Casals J, Piquero-Casals V, la Rotta E, Menta Simonsen Nico M. Crusted scabies in cushingoid child treated with oral ivermectin. Dermatol Argent. 2002;8:136-40.

13. Robert M, Faisant A, Cognet O, Rabodonirina M, Peyron F, Piquemal M, et al. Autochthonous and persistent cutaneous larva migrans in an infant successfully treated by topic albendazole ointment. J Eur Acad Dermatol Venereol. 2019;33(4):e163-4.

14. Romani L, Marks M, Sokana O, Nasi T, Kamoriki B, Wand H, et al. Feasibility and safety of mass drug coadministration with azithromycin and ivermectin for the control of neglected tropical diseases: a single-arm intervention trial. Lancet Glob Health. 2018;6(10):e1132-e8.

15. Sáez-de-Ocariz M, McKinster C, Orozco-Covarrubias L, Tamayo-Sánchez L, Ruiz-Maldonado R. Treatment of 18 children with scabies or cutaneous larva migrans using ivermectin. Clin Exp Dermatol. 2002;27(4):264-7.

16. Soriano-Arandes A, Sulleiro E, Zarzuela F, Ruiz E, Clavería I, Espasa M. Discordances Between Serology and Culture for *Strongyloides* in an Ethiopian Adopted Child With Multiple Parasitic Infections: A Case Report. Medicine (Baltimore). 2016;95(10):e3040.

17. Wimmersberger D, Coulibaly J, Schulz J, Puchkow M, Huwyler J, N'Gbesso Y, et al. Efficacy and Safety of Ivermectin Against *Trichuris trichiura* in Preschool-aged and School-aged Children: A Randomized Controlled Dose-finding Trial. Clin Infect Dis. 2018;67(8):1247-55.

18. Bell T. Treatment of *Pediculus humanus* var. *capitis* infestation in Cowlitz County, Washington, with ivermectin and the LiceMeister comb. Pediatr Infect Dis J. 1998;17(10):923-4.

19. Cahyawari D, Haryati N, Pranata A, Setiabudi D, Pandia V, Shanti M, et al. Successful combination therapy of ivermectin and 5% permethrin cream in recurrent erythroderma caused by crusted scabies. Infections & Infestations Indonesia2015. p. P114.

20. Hall A, Spoerke D, Bronstein A, Kulig K, Rumack B. Human ivermectin exposure. J Emerg Med. 1985;3(3):217-9.

21. Leppard B, Naburi A. The use of ivermectin in controlling an outbreak of scabies in a prison. Br J Dermatol. 2000;143(3):520-3.

22. Macotela-Ruiz E, Méndez Islas C, Nochebuena Ramos E. Tratamiento de escabiasis con Ivermectina por vía oral en una comunidad rural cerrada. Implicaciones epidemiológicas. Dermatología Rev Mex. 1996;40(3 ):179-84.

23. Pannenbecker J, Miller T, Müller J, Jeschke R. Severe capillaria hepatica infestation in a young child. Monatsschr Kinderheilkd. 1990;138(11):767-71.

24. Patel A, Hogan P, Walder B. Crusted scabies in two immunocompromised children: successful treatment with oral ivermectin. Australas J Dermatol. 1999;40(1):37-40.

25. Anderson K, Strowd L. Epidemiology, Diagnosis, and Treatment of Scabies in a Dermatology Office. J Am Board Fam Med. 2017;30(1):78-84.

26. Bota S, Alves P, Constantino C, Maia R. Hypereosinophilia and severe bone disease in an African child: an unexpected diagnosis. BMJ Case Rep. 2019;12(4).

27. Cartel J, Nguyen N, Moulia-Pelat J-P, Plichart R, Martin P, Spiegel A. Mass chemoprophylaxis of lymphatic filariasis with a single dose of ivermectin in a Polynesian community with a high *Wuchereria bancrofi* infection rate. Transactions of the Royal Society of Tropical Medicine and Hygiene 1992;86:537-40.

28. Del Bello D, Viswanathan A, Allegretti G, Cantey P, Tosi M, editors. Case report: four-year-old female from India with nighttime cough, pulmonary infiltrates and an absolute eosinophil count of 53,000. . American Society of Tropical Medicine and Hygiene Meeting; 2014.

29. Fathy F, El-Kasah F, El-Ahwal A. Clinical and parasitological study on scabies in Sirte, Libya. J Egypt Soc Parasitol. 2010;40(3):707-31.

30. Fonseca V, Price H, Jeffries M, Alder S, Hansen R. Crusted scabies misdiagnosed as erythrodermic psoriasis in a 3-year-old girl with down syndrome. Pediatr Dermatol. 2014;31(6):753-4.

31. Garcia E, Goodnough R, Smollin C. A 10-year review of avermectin exposures reported to a poison control system. Clinical Toxicology. 2019;S7(10):870-1052.

32. Ghoshal U, Khanduja S, Chaudhury N, Gangwar D, Ghoshal U. A series on intestinal strongyloidiasis in immunocompetent and immunocompromised hosts. Trop Gastroenterol. 2012;33(2):135-9.

33. González Martínez F, Mellado Peña M, Angulo González de Lara R, García López Hortelano M, Villota Arrieta J, Subirats Fernández MS. Larva Currens as a differential diagnosis of skin lesions in immigrant children. Anales de Pediatría 2010;73 (2):102-4.

34. Hegazy A, Darwish N, Abdel-Hamid I, Hammad S. Epidemiology and control of scabies in an Egyptian village. Int J Dermatol. 1999;38(4):291-5.

35. Huapaya P, Espinoza Y, Huiza A, Sevilla C, Vildósola H. Tratamiento de *Strongyloides stercoralis* con ivermectina y tiabendazole. Anales de la Facultad de Medicina Universidad Nacional Mayor de San Marcos. 2003;64 (2):89-93.

36. Ismail M, Jayakody R. Efficacy of albendazole and its combinations with ivermectin or diethylcarbamazine (DEC) in the treatment of *Trichuris trichiura* infections in Sri Lanka. Ann Trop Med Parasitol. 1999;93(5):501-4.

37. Jibaja E, Blondet L, Obregón Sevillano L, Guillén Ponce R. Ivermectina oral en pediculosis capitis en población infantil. Dermatol Peru. 2003;13(2):113-7.

38. Kan B, Otranto D, Fossen K, Åsbakk K. Dermal swellings and ocular injury after exposure to reindeer. N Engl J Med. 2012;367(25):2456-7.

39. Khalifa R, Abdellatif M, Ahmed A, Yones D, El-Mazary A, Aly L, et al. First case of intestinal acariasis from Egypt. SpringerPlus. 2016;5:e28.

40. Khatoon N, Khan A, Azmi M, Khan A, Shaukat S. Most common body parts infected with scabies in children and its control. Pak J Pharm Sci. 2016;29(5):1715-7.

41. Kienast A, Bialek R, Hoeger P. Cutaneous larva migrans in northern Germany. Eur J Pediatr. 2007;166(11):1183-5.

42. Kokta V. Zoonotic deep cutaneous filariasis--three pediatric cases from Quebec, Canada. Pediatr Dermatol. 2008;25(2):230-2.

43. Kulkarni S, Sayed R, Garg M, Patil V. Neurognathostomiasis in a young child in India: A case report. Parasitol Int. 2015;64(5):342-4.

44. Landehag J, Skogen A, Åsbakk K, Kan B. Human myiasis caused by the reindeer warble fly, *Hypoderma tarandi*, case series from Norway, 2011 to 2016. Euro Surveill. 2017;22(29).

45. Larralde M, Mijelshon L, Gonzalez A, Mora E, Constantakos N. Ivermectin-responsive crusted scabies in four patients. Pediatr Dermatol. 1999;16(1):69-70.

46. Lehmberg J, Roper B, Omoruyi E, Evangelista M. Skin Plaques Mimicking Psoriasis. J Pediatr. 2015;167(4):937-.e1.

47. Marlière V, Roul S, Labrèze C, Taïeb A. Crusted (Norwegian) scabies induced by use of topical corticosteroids and treated successfully with ivermectin. J Pediatr. 1999;135(1):122-4.

48. McLucas P, Fulchiero G, Fernandez E, Miller J, Zaenglein A. Norwegian scabies mimicking onychomycosis and scalp dermatitis in a child with IPEX syndrome. J Am Acad Dermatol. 2007;56(2 Suppl):S48-9.

49. Moulia-Pelat J, Nguyen L, Hascoet H, Luquiaud P, Nicolas L. Advantages of an annual single dose of ivermectin 400 micrograms/kg plus diethylcarbamazine for community treatment of bancroftian filariasis. Trans R Soc Trop Med Hyg. 1995;89(6):682-5.

50. Mushtaq A, Khurshid K, Pal S. Comparison of efficacy and safety of oral ivermectin with topical permethrin in treatment of scabies. J Pakistan Asssoc Dermatologists. 2010;20:227-31.

51. Nguyen N, Moulia-Pelat J, Cartel J. Control of bancroftian filariasis in an endemic area of Polynesia by ivermectin 400 micrograms/kg. Trans R Soc Trop Med Hyg. 1996;90(6):689-91.

52. Oliván-Gonzalvo G. Gnathostomiasis after a trip to China for international adoption. Med Clin (Barc). 2006;126(19):758-9.

53. Roberts L, Huffam S, Walton S, Currie B. Crusted scabies: clinical and immunological findings in seventy-eight patients and a review of the literature. J Infect. 2005;50(5):375-81.

54. Sandoval L, Mercado R, Apt W, Navarrete C, Contreras-Levicoy J, Ueta M, et al. Strongyloidosis no autócotona en Chile . Descripción de un brote familiar. Parasitolo Latinoam. 2004;59:76-8.

55. Shah I, Barot S, Madvariya M. Eosinophilic meningitis: a case series and review of literature of *Angiostrongylus cantonensis* and *Gnathostoma spinigerum*. Indian J Med Microbiol. 2015;33(1):154-8.

56. Ruiz-Maldonado R. Pimecrolimus related crusted scabies in an infant. Pediatr Dermatol. 2006;23(3):299-300.

57. Beeres D, Ravensbergen S, Heidema A, Cornish D, Vonk M, Wijnholds L, et al. Efficacy of ivermectin mass-drug administration to control scabies in asylum seekers in the Netherlands: A retrospective cohort study between January 2014-March 2016. PLoS Negl Trop Dis. 2018;12(5):e0006401.

58. Brodine S, Thomas A, Huang R, Harbertson J, Mehta S, Leake J, et al. Community based parasitic screening and treatment of Sudanese refugees: application and assessment of Centers for Disease Control guidelines. Am J Trop Med Hyg. 2009;80(3):425-30.

59. Burkhart C, Burkhart C. Oral ivermectin therapy for *Phthiriasis palpebrum*. Arch Ophthalmol. 2000;118(1):134-5.

60. Gonzalez C, Galilea N, Pizarro K. Autochthonous cutaneous larva migrans in Chile. A case report. . Revista Chilena de Pediatría. 2015;86:426-9.

61. Haar K, Romani L, Filimone R, Kishore K, Tuicakau M, Koroivueta J, et al. Scabies community prevalence and mass drug administration in two Fijian villages. Int J Dermatol. 2014;53(6):739-45.

62. Marks M, Toloka H, Baker C, Kositz C, Asugeni J, Puiahi E, et al. Randomized Trial of Community Treatment With Azithromycin and Ivermectin Mass Drug Administration for Control of Scabies and Impetigo. Clin Infect Dis. 2019;68(6):927-33.

63. Montour J, Lee D, Snider C, Jentes E, Stauffer W. Absence of *Loa loa* Microfilaremia among Newly Arrived Congolese Refugees in Texas. Am J Trop Med Hyg. 2017;97(6):1833-5.

64. Moser R, Auer H, Prenner-Glas C, Klein G. Transient pruritus in an Ethiopean adoptee in Austria. Wien Klin Wochenschr. 2008;120(19-20 Suppl 4):107-11.

65. Nguyen N, Moulia-Pelat J, Glaziou P, Martin P, Cartel J. Advantages of ivermectin at a single dose of 400 micrograms/kg compared with 100 micrograms/kg for community treatment of lymphatic filariasis in Polynesia. Trans R Soc Trop Med Hyg. 1994;88(4):461-4.

66. Noguera-Morel L, Gerlero P, Hernandez-Martin A, Torrelo A, editors. Retrospective study of ivermectin treatment in 12 patients with rosacea and/or perioral dermatitis. Pediatric Dermatology- 13th Congress of the European Society for Pediatric Dermatology, ESPD 2016.

67. Noguera-Morel L, Gerlero P, Torrelo A, Hernandez-Martin A. Ivermectin therapy for papulopustular rosacea and periorificial dermatitis in children: A series of 15 cases. J Am Acad Dermatol. 2017;76(3):567-70.

68. Pilger D, Khakban A, Heukelbach J, Feldmeier H. Self-diagnosis of active head lice infestation by individuals from an impoverished community: high sensitivity and specificity. Rev Inst Med Trop Sao Paulo. 2008;50(2):121-2.

69. Posey D, Blackburn B, Weinberg M, Flagg E, Ortega L, Wilson M, et al. High prevalence and presumptive treatment of schistosomiasis and strongyloidiasis among African refugees. Clin Infect Dis. 2007;45(10):1310-5.

70. Ro T, Sood A, Kelly K, Morrell D. What Is the Cause of the Chronic Erythematous Scaling Plaques on This 22-Month-Old Girl and Her Family? Clin Pediatr (Phila). 2018;57(7):874-8.

71. Schar F, Hattendorf J, Khieu V, Muth S, Char M, Marti H, et al. *Strongyloides stercoralis* larvae excretion patterns before and after treatment. Parasitology. 2014;141(7):892-7.

72. Vanhaecke C, Perignon A, Monsel G, Regnier S, Bricaire F, Caumes E. The efficacy of single dose ivermectin in the treatment of hookworm related cutaneous larva migrans varies depending on the clinical presentation. J Eur Acad Dermatol Venereol. 2014;28(5):655-7.

73. Veraldi S, Valsecchi M. Imported tungiasis: a report of 19 cases and review of the literature. Int J Dermatol. 2007;46(10):1061-6.

74. Zegpi T, Salomone B, Musalem N. Sarna noruega en lactante. Rev Chilena Dermatol. 2004;20(3):192-5.

75. Bouchaud O, Houze S, Schiemann R, Durand R, Ralaimazava P, Ruggeri C, et al. Cutaneous larva migrans in travelers: a prospective study, with assessment of therapy with ivermectin. Clin Infect Dis. 2000;31(2):493-8.

76. Brooks P, Grace R. Ivermectin is better than benzyl benzoate for childhood scabies in developing countries. J Paediatr Child Health. 2002;38(4):401-4.

77. Eberhard M, Ostovar G, Chundu K, Hobohm D, Feiz-Erfan I, Mathison B, et al. Zoonotic Onchocerca lupi infection in a 22-month-old child in Arizona: first report in the United States and a review of the literature. Am J Trop Med Hyg. 2013;88(3):601-5.

78. Kaminsky R, Reyes-García S, Zambrano L. Unsuspected *Strongyloides stercoralis* infection in hospital patients with comorbidity in need of proper management. BMC Infect Dis. 2016;16:98.

79. Suroshe B, Rathod K, Kulkarni V, Chiklonde R. Scabies in children and its outcome with topical permethrin and oral ivermectin: a single center prospective study. Int J Contemp Pediatr. 2017;4(6):2083-7.

80. Bécourt C, Marguet C, Balguerie X, Joly P. Treatment of scabies with oral ivermectin in 15 infants: a retrospective study on tolerance and efficacy. Br J Dermatol. 2013;169(4):931-3.

81. Bada-del Moral M, Arenas R, del Pilar Bada-Pérez M, González-Ramirez M, Vergara-Takahashi L. Trombidiasis ("tlazahuate") en Veracruz, Mexico. Dermatol Rev Mexicana. 2015;59:233-7.

82. Ordóñez L, Angulo E. Eficacia de la ivermectina en el tratamiento de ninos colombianos parasitados por *Strongyloides stercoralis*. Biomédica. 2004;24:33-41.

83. Van den Enden E, Stevens A, Van Gompel A. Treatment of cutaneous larva migrans. N Engl J Med. 1998;339(17):1246-7.

84. Alipour H, Goldust M. The efficacy of oral ivermectin vs. sulfur 10% ointment for the treatment of scabies. Ann Parasitol. 2015;61(2):79-84.

85. Cunningham C, Kazacos K, McMillan J, Lucas J, McAuley J, Wozniak E, et al. Diagnosis and management of *Baylisascaris procyonis* infection in an infant with nonfatal meningoencephalitis. Clin Infect Dis. 1994;18(6):868-72.

86. Goldust M, Rezaee E. Comparative trial of oral ivermectin versus sulfur 8% ointment for the treatment of scabies. J Cutan Med Surg. 2013;17(5):299-300.

87. Goldust M, Rezaee E, Hemayat S. Treatment of scabies: Comparison of permethrin 5% versus ivermectin. J Dermatol. 2012;39(6):545-7.

88. Goldust M, Rezaee E, Raghifar R. Comparison of oral ivermectin versus crotamiton 10% cream in the treatment of scabies. Cutan Ocul Toxicol. 2014;33(4):333-6.

89. Goldust M, Rezaee E, Raghifar R, Hemayat S. Comparing the efficacy of oral ivermectin vs malathion 0.5% lotion for the treatment of scabies. Skinmed. 2014;12(5):284-7.

90. Goldust M, Rezaee E, Raghifar R, Naghavi-Behzad M. Ivermectin vs. lindane in the treatment of scabies. Ann Parasitol. 2013;59(1):37-41.

91. Mohebbipour A, Saleh P, Goldust M, Amirnia M, Zadeh Y, Mohamadi R, et al. Treatment of scabies: comparison of ivermectin vs. lindane lotion 1%. Acta Dermatovenerol Croat. 2012;20(4):251-5.

92. Mohebbipour A, Saleh P, Goldust M, Amirnia M, Zadeh Y, Mohamad R, et al. Comparison of oral ivermectin vs. lindane lotion 1% for the treatment of scabies. Clin Exp Dermatol. 2013;38(7):719-23.

93. Ranjkesh M, Naghili B, Goldust M, Rezaee E. The efficacy of permethrin 5% vs. oral ivermectin for the treatment of scabies. Ann Parasitol. 2013;59(4):189-94.

94. Victoria J. Ivermectina en pediculosis capitis. Act Terap Dermatol 1998;21:448-51.

95. Victoria J. Uso de ivermectina en niños. Dermatol Pediatr. 2003;1:61-5.

96. Victoria J, Ahumada N, González F. Pediculosis Capitis: Tratamiento de 100 ninos con Ivermectina. Act Terap Dermatol. 1997(20):99-103.

97. Kobylinski K, von Seidlein L. Ivermectin Safety in Small Children (NCT04332068). University of Oxford; 2020.
