# Supplementary material for: A systematic review and an individual patient data meta-analysis of ivermectin use in children weighing less than fifteen kilograms: Is it time to reconsider the current contraindication?
Source: PLoS Negl Trop Dis. 2021 Mar 17;15(3):e0009144. doi: 10.1371/journal.pntd.0009144 (PMC7968658; doi:10.1371/journal.pntd.0009144)
Supplement: S2 Table — Number and classification of adverse events reported for each study included in the systematic analysis. (DOCX) [file pntd.0009144.s003.docx]

**S2 Table. Summary of adverse events reported by study.**

|  | | **Children with AE** | | | **AE Reported** | | | | | | | |
| --- | --- | --- | --- | --- | --- | --- | --- | --- | --- | --- | --- | --- |
| **Study ID** | **N evaluated** | **N** | **%** | **95% CI** | **Diarrhoea** | **Eczema** | **Headache** | **Itching** | **Joint**  **Pain** | **Stomach**  **Ache** | **Vomiting** | **Symm**  **Edema** |
| 1 | 17 | 0 | 0.00% | 0.00% - 19.61% |  |  |  |  |  |  |  |  |
| 3 | 1 | 0 |  |  |  |  |  |  |  |  |  |  |
| 4 | 1 | 0 |  |  |  |  |  |  |  |  |  |  |
| 5 | 1 | 1 |  |  | 0 | 0 | 0 | 0 | 0 | 0 | 0 | 1 |
| 6 | 1 | 0 |  |  |  |  |  |  |  |  |  |  |
| 7 | 1 | 0 |  |  |  |  |  |  |  |  |  |  |
| 8 | 1 | 0 |  |  |  |  |  |  |  |  |  |  |
| 9 | 4 | 0 |  |  |  |  |  |  |  |  |  |  |
| 10 | 1 | 0 |  |  |  |  |  |  |  |  |  |  |
| 11 | 4 | 0 |  |  |  |  |  |  |  |  |  |  |
| 12 | 169 | 7 | 4.14% | 1.68% - 8.35% | 1 | 5 | 0 | 0 | 0 | 0 | 1 | 0 |
| 13 | 838 | 2 | 0.24% | 0.03% - 0.86% | 1 | 0 | 0 | 0 | 1 | 0 | 0 | 0 |
| 14 | 44 | 5 | 11.36% | 3.79% - 24.56% | 2 | 0 | 2 | 2 | 0 | 1 | 1 | 0 |
| 15 | 4 | 0 |  |  |  |  |  |  |  |  |  |  |
| 17 | 1 | 0 |  |  |  |  |  |  |  |  |  |  |
